# Myelopoietic Efficacy of Orlistat in Murine Hosts Bearing T Cell Lymphoma: Implication in Macrophage Differentiation and Activation

**DOI:** 10.1371/journal.pone.0082396

**Published:** 2013-12-03

**Authors:** Shiva Kant, Ajay Kumar, Sukh Mahendra Singh

**Affiliations:** School of Biotechnology, Banaras Hindu University, Varanasi, India; European Institute of Oncology, Italy

## Abstract

Orlistat, an inhibitor of fatty acid synthase (FASN), acts as an antitumor agent by blocking *de novo* fatty acid synthesis of tumor cells. Although, myelopoiesis also depends on *de novo* fatty acid synthesis, the effect of orlistat on differentiation of macrophages, which play a central role in host’s antitumor defence, remains unexplored in a tumor-bearing host. Therefore, the present investigation was undertaken to examine the effect of orlistat administration on macrophage differentiation in a T cell lymphoma bearing host. Administration of orlistat (240 mg/kg/day/mice) to tumor-bearing mice resulted in a decline of tumor load accompanied by an augmentation of bone marrow cellularity and survival of bone marrow cells (BMC). The expression of apoptosis regulatory caspase-3, Bax and Bcl2 was modulated in the BMC of orlistat-administered tumor-bearing mice. Orlistat administration also resulted in an increase in serum level of IFN-γ along with decreased TGF-β and IL-10. BMC of orlistat-administered tumor-bearing mice showed augmented differentiation into macrophages accompanied by enhanced expression of macrophage colony stimulating factor (M-CSF) and its receptor (M-CSFR). The macrophages differentiated from BMC of orlistat-administered mice showed characteristic features of M_1_ macrophage phenotype confirmed by expression of CD11c, TLR-2, generation of reactive oxygen species, phagocytosis, tumor cell cytotoxicity, production of IL-1,TNF-α and nitric oxide. These novel findings indicate that orlistat could be useful to support myelopoesis in a tumor-bearing host.

## Introduction

Sustained myelopoiesis considered essential to overcome myelosupression in tumor-bearing hosts associated with tumor progression and chemotherapeutic applications [1–3]. Fatty acid synthase (FASN)-dependent *de novo* fatty acid synthesis is identified as an indispensable necessity of hematopoiesis, differentiation and activation of macrophages (Mϕ), which play a central role in host’s antitumor defense [4–9]. Further, the involvement of FASN in M_1_/M_2_ macrophage polarization, expression of TLRs, IL-1, TNF-α and phagocytosis has been reported [4–9]. Moreover, inhibition of FASN alters endotoxin responsiveness of macrophages [9]. Interestingly, FASN requirement has been demonstrated to vary depending on stages of macrophage differentiation [7]. Thus FASN plays an essential role in macrophage differentiation and activation.

FASN dependent de novo fatty acid synthesis is a ubiquitous necessity of transformed cells for membrane biosynthesis [10–17]. Consequently, one of the upcoming anticancer chemotherapeutic regimens depends on inhibition of FASN [10–14,16,17]. We and others have demonstrated that exposure of tumor cells to orlistat, a FASN inhibitor; can manifests tumor-specific cytotoxicity [18–22]. Moreover, impact of FASN inhibition on cell survival displays cell-specific variations [8]. Reports indicate that FASN inhibition arrests membrane-associated functions of macrophages and their differentiation from monocytes [7]. However, to the best of our knowledge there is no report regarding the action of orlistat on myelopoietic differentiation of macrophages in tumor-bearing hosts. Thus in the present study using a murine model of transplantable T cell lymphoma, designated as Dalton’s lymphoma (DL) [20,23–30], we investigated the effect of orlistat administration on bone marrow homeostasis with reference to differentiation and antitumor activation of macrophages. DL originated in the thymus of DBA [H2^d^] strain of mice as thyoma [31,32] and can be transplanted in syngenic mice. Our results demonstrate that orlistat administration to the tumor-bearing hosts can augment myelopoietic differentiation of tumoricidal macrophages.

## Materials and Methods

### 1: Mice and tumor system

Pathogen-free inbred adult mice of BALB/c (H-2d) strain were used at 8-12 weeks of age. The mice were procured from the animal house facility of the Banaras Hindu University approved by the central animal ethical committee and kept in the animal rooms of the School of Biotechnology. The work contained in this manuscript was approved by central animal ethical committee of Banaras Hindu University. The mice received food and water *ad libitum* and were treated with utmost humane care. Dalton’s lymphoma (DL) is maintained in ascitic form by serial transplantation in BALB/c mice or in an in vitro cell culture system by serial passage. Irrespective of whether the DL cells were obtained from the in vitro culture system maintained as suspension cultures or from the ascitic fluid they exhibited similar phenotypic features. Serial passage of DL in mice was carried out by transplanting 1x 10^5^ DL cells mouse^-1^ in 0.5 ml phosphate buffered saline (PBS) as standardised previously in our laboratory [33]

### 2: Reagents

All reagents used were of tissue culture or analytical grade. Tissue culture medium RPMI 1640 was purchased from Hyclone (USA), supplemented with 20 mg/ml gentamycin, 100 mg/ml streptomycin, 100 IU penicillin purchased from Himedia (India) and 10 % fetal calf serum from Hyclone (USA). Antibodies against Bcl2, Caspase-3, Bax, IL-1, IL-6, IL-10, IFN-γ, TNF-α, TLR-2, TGF-β & β-actin and fluorochrome conjugated antibodies against F4/80, CD11c and their isotype controls were obtained from Sigma-Alderich (USA), Imgenex (USA), BD Pharmingen (USA), eBioscience (USA) and Chemicon (UK). Secondary antibodies conjugated to alkaline phosphatase were obtained from Bangalore Genie (India). Primers for RT-PCR (Table. 1) were purchased from Hysel, India. BCIP/NBT was purchased from Amresco (USA). TUNEL assay kit was purchased from Invitrogen (USA). 

**Table 1:** Primers for RT-PCR.

**Name of Gene**	**Primer sequence**
M-CSF	F-5’-CGGGCATCATCCTAGTCTTGCTGACTGTT-3’
	R-5’-AAATAGTGGCAGTATGTGGGGGGCATCCT-3’
M-CSFR	F-5’-TCATTCAGAGCCAGCTGCCCAT-3’
	R-5’-ACAGGCTCCCAAGAGGTTGACT-3’
β-actin	F-5’-GGCACAGTGTGGGTGAC-3’
	R-5’-CTGGCACCACACCTTCTAC-3’

### 3: Protocol for orlistat administration to tumor-bearing mice

**Figure 1:**
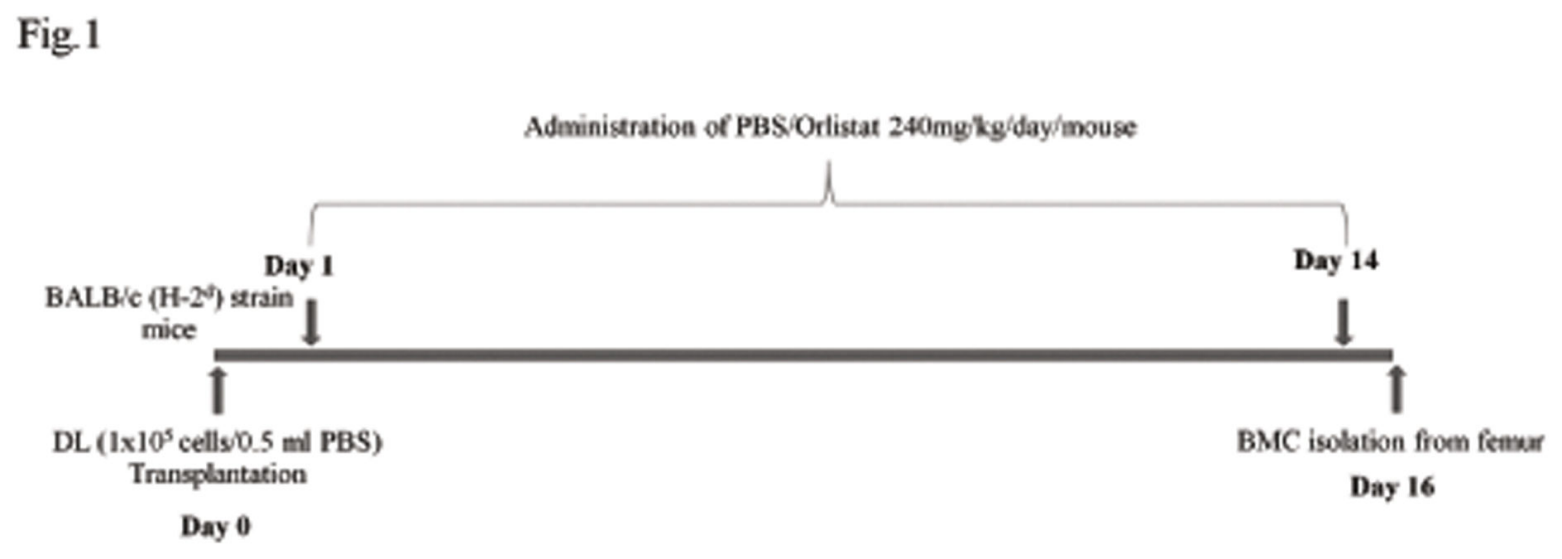
Protocol for administering orlistat to tumor-bearing mice. Mice were transplanted DL cells (1x10^5^cells/0.5 ml PBS) on day 0 following administration of Vehicle alone (control) or containing orlistat 240mg/kg body weight/day up to day 14 post tumor transplantation. On day 16 BMC were harvested from femurs.

Orlistat was solubilised and administered to tumor-bearing mice in group of nine each (Fig.1), following a protocol described by Kridel et al [21] at a dose of 240mg/kg/day body weight, reported for therapeutic effect [21,34]. Tumor and bone marrow cells were harvested on day 16 following tumor transplantation, which was pre-standardised in preliminary experiments, representing the full blown tumor bearing stage [35], beyond which necrosis of tumor is initiated. Thus, at this stage tumor growth and its ramifications can be conveniently analysed. The number of tumor-associated macrophages (TAM) was determined by flow cytometry of peritoneal exudates cells using Guava Incyte (USA) flow cytometer.

### 4: Bone marrow cell preparation

Bone marrow cell (BMC) were obtained from the femurs of DL-bearing mice following a method described earlier [29]. Briefly, the BMC were obtained from the femoral shafts by flushing with serum-free medium and agitating gently to prepare a single cell suspension. The BMC suspension was washed twice with serum-free medium by centrifugation at 200xg at 4°C. Viability of BMC was estimated using the standard trypan blue dye exclusion test as described previously [24]. Cell suspension was mixed with an equal volume of 0.4% trypan blue in PBS and the cells were counted using a hemocytometer. Cells that did not exclude the trypan blue were considered nonviable. Standard Leishman Staining was performed to examine subpopulations of bone marrow cells.

### 5: MTT assay

Cell survival was determined by MTT assay according to a method described earlier [20] with slight modifications. MTT [3-(4,5-dimethylthiazol-2yl)-2,5-diphenyl tetrazolium bromide] (5 mg/ml in PBS) was added to each well (50 μl/well) of the culture plate containing 200 ml medium and incubated at 37°C for 4 h. The medium was then carefully removed, without disturbing the dark blue formazan crystals. Fifty μl DMSO was added to each well and mixed thoroughly to dissolve the formazan crystals. Plates were then read on a microplate reader (Labsystems, Finland) at a wavelength of 540 nm. 

### 6: Preparation of L929-conditioned medium

L929-cell conditioned medium (L929CM) was used as a source of macrophage-colony stimulating factor (M-CSF) [36]. L929CM was prepared according to a method described earlier [29]. L929 cells, obtained from National Centre for Cell Science, Pune, India, were incubated in RPMI-1640 supplemented with 10% FCS to achieve exponential growth. Cell-free supernatant was then harvested from the confluent monolayer of L929 cells, passed through 0.22 mm membrane filter and kept at -20°C until use.

### 7: Wright Giemsa staining

Apoptotic cell population was enumerated by Wright Giemsa as described earlier [37]. Cell suspension was smeared on a slide, air dried, fixed in methanol, stained with Wright Giemsa staining solution, mounted on glycerine and analyzed under light microscope (Carl Zeiss, Germany) at 400x magnification. Apoptotic cells were identified on the basis of morphological features that included contracted cell bodies, condensed, uniformly circumscribed and densely stained chromatin, and membrane bound apoptotic bodies containing one or more nuclear fragments. The percentage of apoptotic cells was determined by counting more than 300 cells in at least three separate randomly selected microscopic fields.

### 8: TUNEL assay

Apoptotic cells were identified by TUNEL assay kit following the manufacturer’s instructions as described earlier [23]. Briefly, cells were fixed in 1% (w/v) paraformaldehyde solution in PBS at 4°C for 15 min followed by incubation in 70% ethanol at -20°C for 30 min. Cells were then incubated in DNA labeling solution containing TdT enzyme and BrdUTP at 37°C for 60 min followed by washing with rinse buffer and incubation in Alexa Fluor 488 dye-labeled anti-BrdU antibody for 30 min at room temperature. Apoptotic cells were identified both under phase contrast and fluorescence optics. Cells which fluoresced brightly were apoptotic when observed under fluorescence optics of fluorescence microscope (Nikon, Japan). The percentage of apoptotic cells was determined by counting more than 300 cells in at least three separate randomly selected microscopic fields.

### 9: Bone marrow colony assay

Bone marrow colonies were prepared according to a method described earlier using culture medium containing methylcellulose [29]. Briefly, BMC (1x10^4^cells/ml) were suspended in a mixture containing 0.9% (w/v) methylcellulose with 30% (v/v) FCS and 20% (v/v) L929CM. The mixture was gently vortexed, plated in a 35 mm plastic culture dish (Greiner, Germany) and incubated at 37°C in a humidified atmosphere of 5% CO_2_ in air for 10 days. Bone marrow colonies were counted after ten days of incubation. An aggregate of more than 25 cells was counted as a single colony-forming unit (CFU). Colonies of different types were identified on the basis of their morphological features. Those with macrophage-like morphology were designated as CFU-M, granulocyte-macrophage morphology as CFU-GM and granulocyte morphology as CFU-G.

### 10: Culture and isolation of bone marrow-derived macrophages (BMDM)

BMDM were obtained as described previously [28]. Briefly, BMC were flushed from femoral shafts with chilled serum-free medium. A single cell suspension of BMC was prepared and incubated in plastic tissue culture flasks for 2 h to remove adherent bone marrow macrophages. The non-adherent BMC (2.5 x10^6^cells/ml) were incubated for 10 days in medium containing L929CM (20% v/v). The BMDM thus obtained were scrapped by cell scrapper and replated in 96 well or 6 well tissue culture plates at a density of 2.5x10^5^ cells/ml and further incubated for 24 h in medium alone or containing IFN-γ (100 IU/ml) plus LPS (10 ng/ml). After 24 h of incubation the cell-free culture supernatant was harvested for ELISA and the cells were used for other estimations described below. BMDM cultivated in 6 well tissue culture plates containing glass cover slips were used for estimating phagocytosis and immune-fluorescence staining.

### 11: Cytotoxicity assay

$\begin{array}{c} \%\;cytotoxicity=\left(O.D.of\;\;DL\;\;cells\;\;incubated\;\;alone+O.D.\;\;of\;\;BMDM\;\;incubated\;\;alone\right)-O.D.\;of\;\;DL\;\;cells\;\;incubated\;\;with\;\;BMDM\\ \;\;\;\;\;\;\_\_\_\_\_\_\_\_\_\_\_\_\_\_\_\_\_\_\_\_\_\_\_\_\_\_\_\_\_\_\_\_\_\_\_\_\_\_\_\_\_\_\_\_\_\_\_\_\_\_\_\_\_\_\_\_\_\_\_\_\_\_\_\_\_\_\_\_\_\_\_\_\_\_\_\_\_\_\_\_\_\_\_\_\_\_\_\_\_\_\_\_\_\_\_\_\_\_\_\_\_\_\_\_\_\_\_\_\_X100\\ O.D.\;of\;\;DL\;\;cells\;\;incubated\;\;alone+O.D.of\;\;BMDM\;\;incubated\;\;alone \end{array}$

Macrophagemediated tumor cytotoxicity was assayed by measuring the killing of target DL cells as described earlier with some modifications using MTT [25] and standard LDH release assay following the procedure described by Naama et al. [38]. Tumor cells were co-incubated with BMDM at an effecter to target cell ratio of 10:1. In MTT method, after 48 h, the incubation was terminated followed by addition of 50 μl of MTT solution to the co-culture of BMDM and tumor cells to determine cell survival as described above. Percent cytotoxicity was calculated by the following formula.

$\begin{array}{c} \%\;LDH\;release=\;O.D.\;\left[\exp erimental\right]-O.D.\;\left[Spon\tan eous\right]\\ \;\;\;\;\;\;\;\;\;\_\_\_\_\_\_\_\_\_\_\_\_\_\_\_\_\_\_\_\_\_\_\_\_\_\_\_\_\_\_\_\_\_\_\_\_\_\_\_\_\_\_X100\\ \;\;\;\;\;O.D.\;\left[Maximum\right] \end{array}$

In LDH release method following coincubation of BMDM with tumor cells LDH release was estimated by measuring the end product NAD^+^, generated from NADH and pyruvate by the catalytic action of LDH. O.D. was measured at 340nm. % cytotoxicity was calculated by the following formula.

Experimental = BMDM plus Tumor cells
Spontaneous = Tumor cells alone
Maximum = Lysate of tumor cells

### 12: Phagocytosis assay

Phagocytic activity of BMDM was carried out as described earlier [27]. BMDM grown on glass cover slips were incubated with heat-killed yeast cells (2×10^8^/ml) for 90 min at 37°C in a CO_2_ incubator. The non-phagocytosed yeast cells were washed with warm PBS thrice. Cells were then fixed in methanol for 2 min. and stained with Giemsa stain for 1 h. Excess stain was washed out under tap water. The cover slips were mounted on a slide and phagocytosis was examined under light microscope.

### 13: ELISA

A standard ELISA was performed to detect the presence of indicated cytokines in sera of control and orlistat administered tumor-bearing mice and BMDM culture supernatent following a method described earlier [20]. Briefly, polystyrene microwell plates (Tarsons, Kolkata, India) were coated with 10 μg of protein sample and incubated overnight at 4°C. In the negative control, test samples were not added to wells of ELISA plates and were processed for subsequent steps in the same way as described for the experimental sets. The plates were washed with 0.15 M PBS containing 0.1% (v/v) Tween 20 (PBS-Tween). Unbound sites were saturated with PBS containing 1% bovine serum albumin (BSA). The plates were again washed with PBS-Tween followed by addition of antibodies against the indicated proteins at a dilution of 1:1000. The plates were incubated at 37°C for 60 min followed by addition of 50 μl of p-nitrophenyl phosphate (NPP) (1 mg/ml) in enzyme substrate buffer. The absorbance was measured after 10 min at 405 nm in an ELISA plate reader (Labsystems, Finland) and the concentration of cytokines is presented as pg/ml. 

### 14: Western immunoblot analysis

Western immunoblot analysis for detection of indicated proteins in BMC was carried out following a method described earlier [37]. BMC were washed with chilled PBS and lysed in 50 µl of lysis buffer (20mM Tris-Cl, pH 8.0, 137 mM NaCl, 10% (v/v) glycerol, 1% (v/v) Triton X-100, 2 mM EDTA; 1 mM phenylmethylsulfonyl fluoride, 20 μM leupeptin containing aprotinin at 0.15 U ml^-1^) for 20 min at 4°C. Protein content in each sample was determined by using standard Bradford method. Twenty µg of Triton X-100 solubilized proteins was separated on 10% SDS-polyacrylamide gel at 20 mA. The gel was processed further for immunoblotting. The separated proteins were transferred onto a nitrocellulose membrane (Sartorius, Germany) (1.5 h at 150 mA), immunoblotted with antibodies against indicated proteins and probed with a secondary antibody: anti-rabbit IgG conjugated to alkaline phosphatase and detected by a BCIP/NBT solution (Amresco, USA). Equal loading of proteins was determined by using equal cell number for preparation of lysates, loading of equal protein content and immunoblotting of β-actin.

### 15: RT–PCR for expression of mRNA

RT–PCR analysis for the expression of mRNA of macrophage colony stimulating factors (M-CSF) and its receptor (MCSF-R) in BMC was carried out according to a method described earlier [23] using a one step RT–PCR cell to cDNA kit (Ambion, USA). Primer sequences for various genes are shown in Table 1. PCR was performed for 15 min to make cDNA at 50°C. The amplification was carried out for 30 cycles with initial denaturation at 94°C for 2 min followed by annealing (annealing temperature as per respective primer design) for 30 sec and elongation at 72°C for 30 sec. The samples were separated on an agarose gel (1%) containing ethidium bromide (0.3 μg/ml). Bands were visualized and analyzed on a UV-transilluminator (Biorad, Australia). 

### 16: Measurement of intracellular reactive oxygen species (ROS)

ROS measurement was carried out as described by Furuta et al [39] with slight modifications. BMDM (1x10^6^ cells/ml) of control and orlistat groups were washed followed by incubation with HBSS containing the fluorescent dye dichlorodihydrofluorescein diacetate, (DCFDA) at a final concentration 0.1mM. The cells were further incubated at 37°C for 45 min, followed by washing with PBS. The cells stained with the dye were visualized under fluorescence microscope (Nikon, Japan) at a magnification of 400× and photographed. 

### 17: Immunofluorescent staining

Immunofluorescence staining of bone marrow derived macrophage (BMDM) was carried out using a protocol described earlier [25] with slight modification. Briefly, BMDM grown on cover slips were incubated in PBS containing FITC conjugated anti-F4/80, TLR-2 or PE conjugated anti-CD11c for 30 min at 37 °C. After incubation, cells were washed with PBS and fixed in a mixture of acetic acid and ethanol (5:95) for 10 min at -10°C. Cells were then observed under fluorescence microscope (Nikon, Japan) in at least four randomly selected microscopic fields at 400x magnification.

### 18: Nitrite assay

The concentration of stable nitrite NO_2_^-^, the end product from NO generation, was determined by the method described earlier on the Griess reaction [23]. Test samples were incubated with an equal volume of Griess reagent [1 part of 1% (w/v) sulfanilamide in 2.5% H_3_PO_4_ plus 1 part of 0.1% (w/v) naphthyl ethylene diaminedihydrochloride; two parts being mixed together within 12 h of use and kept chilled] at room temperature for 10 min in a 96-well microtiter plate. The absorbance at 540 nm was determined with an automatic ELISA plate reader (Labsystem, Finland). Nitrite content was quantified by extrapolation from a standard curve of NaNO_2_ in each experiment. In all the experiments nitrite content in the wells containing medium without cells was also measured and subtracted.

### 19: Statistical analysis

Experiments were conducted thrice in triplicate. The statistical significance of differences between test groups was analyzed by Student’s *t* test. The difference was considered significant when *p* was less than 0.05.

## Results

### 1: Orlistat administration to tumor-bearing mice augments bone marrow Cellularity

**Figure 2:**
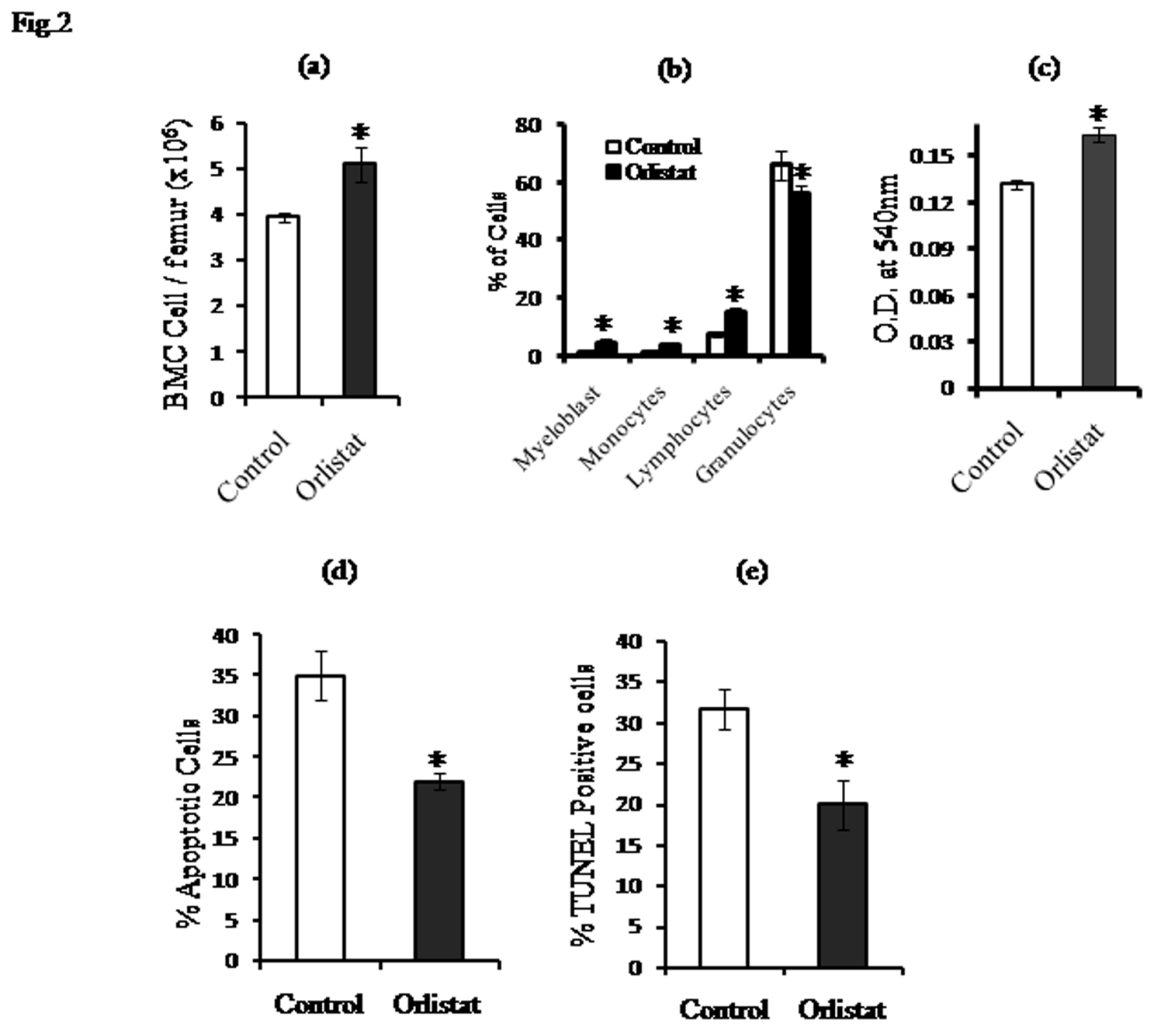
Effect of orlistat administration to tumor-bearing mice on BMC count, survival and apoptotic population. Viable cells in BMC harvested from the femur of control and orlistat-administered tumor-bearing mice were enumerated by trypan blue dye exclusion test (a). A differential count of the BMC population was performed by Leishman staining (b). BMC (1x10^6^ cells/ml) of control and orlistat-administered groups were incubated for 24h in 96 well culture plates followed by estimation of cell survival by MTT assay (c). Induction of apoptosis was estimated by Wright Giemsa staining (d) and TUNEL assay (e). Values are mean ± SD of three independent experiments done in triplicate.* *p<0.05 vs*. values of respective control.

Since there is no report regarding the effect of orlistat administration to tumor-bearing hosts on bone marrow homeostasis, we investigated if orlistat administration to tumor-bearing mice can alter bone marrow cellularity. As shown in Figure 2a, the number of viable BMC was significantly higher in orlistat-administered tumor-bearing mice compared to untreated control. Further to investigate if the observed augmentation of bone marrow cellularity is associated with an altered cell survival, BMC (1x10^6^ cells/ml) harvested from control and orlistat-administered tumor-bearing hosts were cultured for 24 h in 96 well culture plates followed by MTT assay for estimating cell survival (Figure 2c) or processed for enumeration of apoptotic population by Wright Giemsa staining (Figure 2d) and TUNEL assays (Figure 2e). BMC obtained from orlistat-administered tumor-bearing hosts showed a significantly augmented cell survival accompanied by a declined in apoptotic population compared to respective controls. We also examined the effect of orlistat administration on different subpopulations of BMC. As shown in Figure 2b, differential count of BMC harvested from control and orlistat administered tumor-bearing hosts revealed a significant increase in the count of myeloblasts and monocytes compared to control, indicating that orlistat administration augmented the differentiation of macrophage precursors.

**Figure 3:**
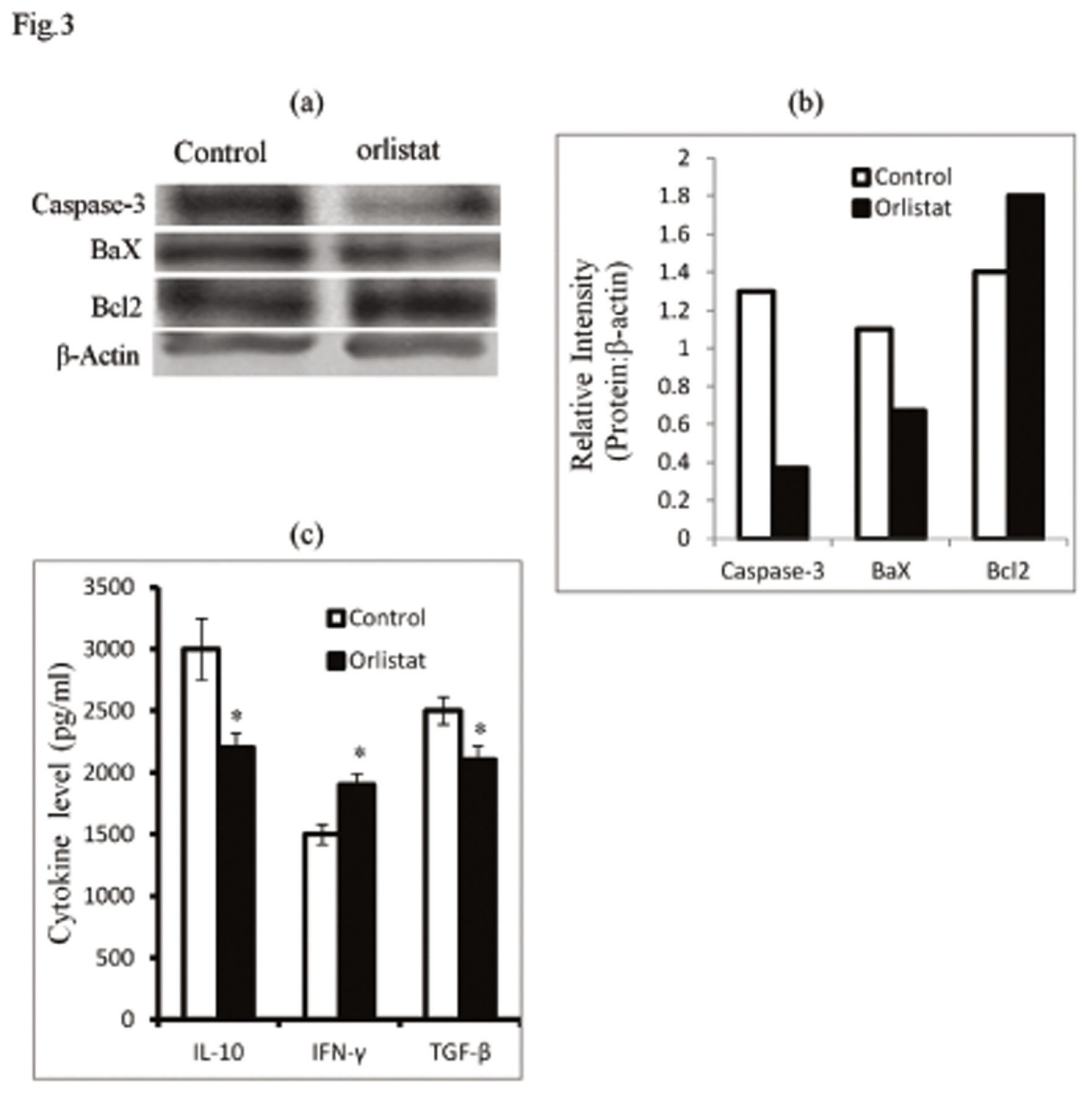
Orlistat administration to tumor-bearing mice alters the expression pattern of cell survival regulatory molecules. Cell lysates of BMC (1x10^6^ cells/ml) harvested from control and orlistat-administered tumor bearing mice were processed for western blot analysis to examine expression pattern of the indicated cell survival regulatory proteins (a,b). Bars shown in (b) are the densitometric scan of bands shown in (a). Data shown is from a representative experiment out of three independent experiments with similar results. Sera harvested from control and orlistat-administered tumor-bearing mice on day 16 post-tumor transplantation were immunodetected by ELISA for the level of the indicated cytokines (c). Values in (c) are mean ± SD of three independent experiments done in triplicate.*p<0.*05* *vs*. values of respective control.

In order to understand the molecular mechanism(s) of orlistat-dependent augmentation of BMC survival, we checked the expression pattern of some key cell survival regulatory molecules. As shown in Figure 3 (a, b) BMC of orlistat-administered tumor-bearing hosts showed an inhibition in the expression of pro-apoptotic Caspase-3 and Bax proteins accompanied by an increased expression of anti-apoptotic Bcl2. In order to explore the underlying regulatory mechanism(s), sera obtained from control and orlistat-administered tumor-bearing mice were analysed for the level of IL-10, TGF-β and IFN-γ, which are reported to regulate BMC survival and differentiation of macrophage precursors [40–45]. Results are shown in Figure 3c. Level of TGF-β and IL-10 significantly declined accompanied by an elevation of IFN-γ in the serum of orlistat-administered tumor-bearing mice compared to respective controls.

### 2: Orlistat administration to tumor-bearing hosts augments myelopoiesis

**Figure 4:**
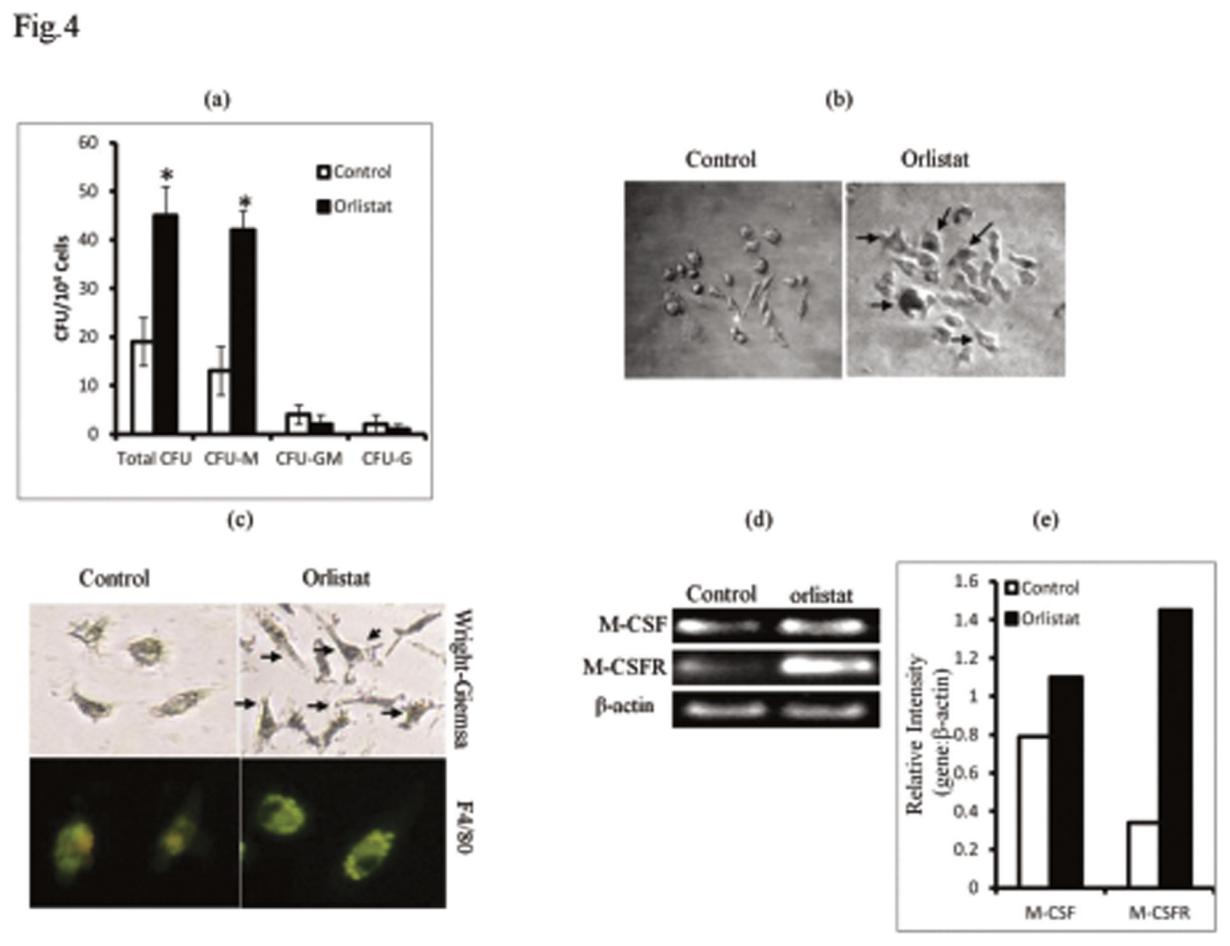
Orlistat augments differentiation of BMDM associated with increased expression of M-CSF and M-CSFR. BMC (1x10^4^ cells/ml) harvested from control or orlistat-administered tumor-bearing mice were cultured in vitro in the presence of L929 culture medium (20%v/v) as a source of M-CSF, for 10 days to allow the BMC to differentiate into colonies. Colonies were counted based on cellular morphology of each colony forming unit (CFU) displaying features of CFU-M, CFU-GM and CFU-G phenotype (a). CFU-M obtained from the BMC of control group displayed lesser number of Mϕ-like cells compared to orlistat-treated group where the colonies were denser with larger macrophage like cells, as indicated by arrows (b). BMC (1x10^6^ cells/ml) obtained from control or orlistat-administered tumor-bearing mice were also processed for RT-PCR to detect expression of mRNA for MCSF and M-CSFR. Bars shown in (e) are densitometric scan of bands shown in (d), which are from a representative experiments out of 3 experiments with similar results. BMDM grown on glass cover slips in petri-dishes were stained with Wright Giemsa stain (c upper panel) and F4/80 FITC-conjugated antibody (c lower panel). As indicated by arrows BMDM of orlistat administered group showed increased size, spreading and cytoplasmic extensions. Plates shown are from a representative experiment. Values shown in (a) are mean ± SD of three independent experiments done in triplicate.*p<0.*05* *vs*. values of respective control.

Since, we observed an improved BMC survival following *in vivo* administration of orlistat to tumor-bearing hosts, in the next part of the investigation we checked if it was also associated with modulation of myelopoesis. BMC harvested from control and orlistat-administered tumor-bearing mice were allowed to differentiate in macrophage lineage in response to M-CSF *in vitro*. Results are shown in Figure 4. Orlistat administration resulted in a significant increase in the count of CFU-M (Figure 4a) displaying larger size compared to control (Figure 4b). BMDM, which were determined to be F4/80+ve, showed a better macrophage spreading with longer cytoplasmic extensions in orlistat group compared to control (Figure 4c). As BMC of orlistat-administered tumor-bearing mice displayed an increased responsiveness to M-CSF for differentiation in Mϕ lineage, we examined if these BMC also showed an altered expression of M-CSF and M-CSFR genes. BMC of orlistat-administered tumor-bearing mice showed an increased expression of M-CSF and M-CSFR compared to control (Figure 4d,e).

### 3: Effect of orlistat administration to tumor-bearing mice on induction of tumor cell apoptosis

**Figure 5:**
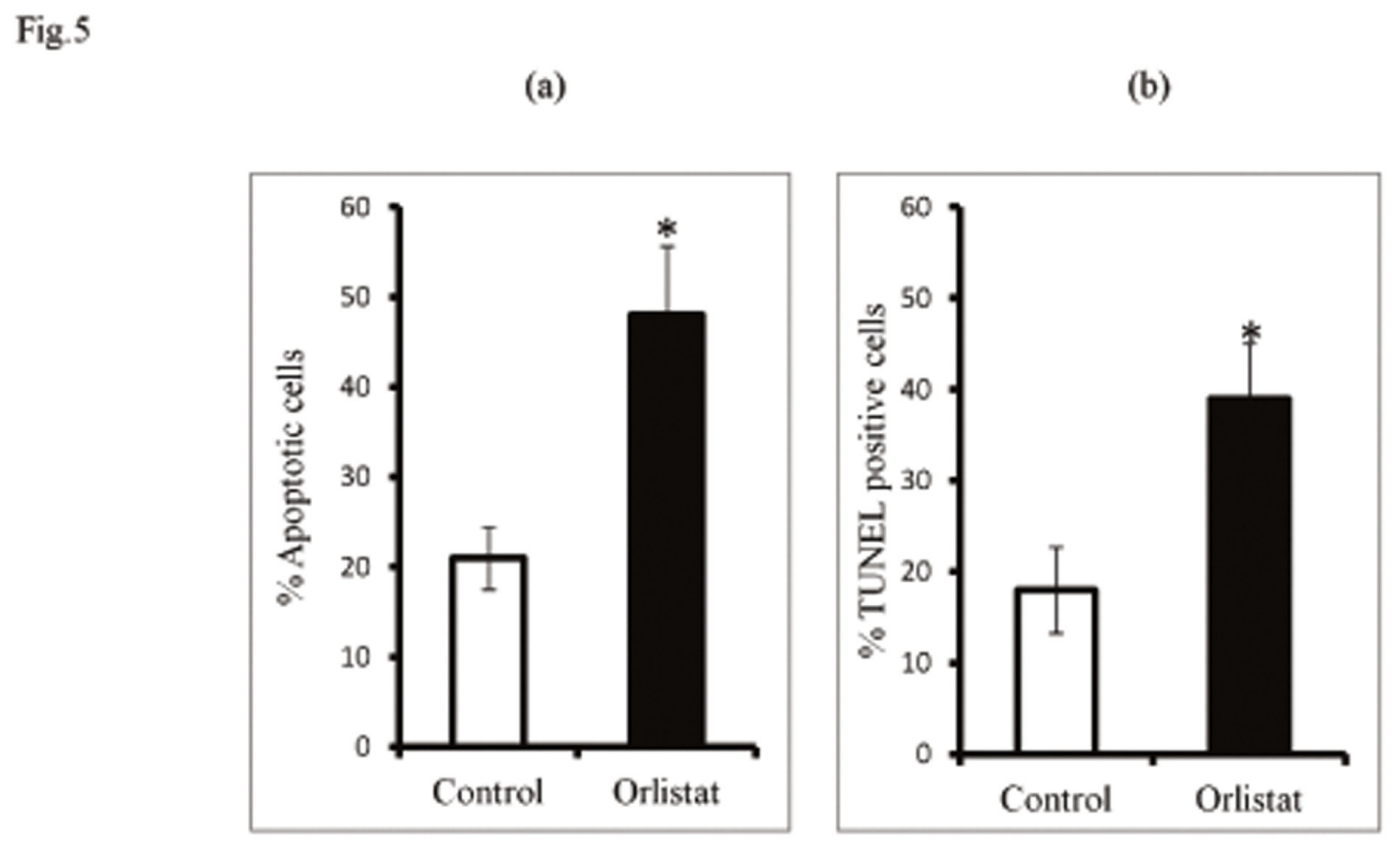
Effect of orlistat administration to tumor-bearing mice on apoptotic tumor cell population. Tumor cells (1x10^6^ cells/ml) harvested from control and orlistat administered tumor-bearing mice were analysed for apoptotic cells by Wright-Giemsa (a) and TUNEL assay (b). Values shown in (a) & (b) are mean ± SD of three independent experiments done in triplicate.*p<0.*05* *vs*. values of respective control.

In order to assess the antitumor potential of orlistat, tumor-bearing mice were administered with vehicle alone (control) or containing orlistat as described in material and methods. Tumor cells were harvested on day 16 post tumor transplantation followed by enumeration of apoptotic tumor cells by Wright-Giemsa staining and TUNEL assay. Results are shown in Figure 5. Orlistat administration to tumor-bearing mice resulted in a significantly augmented population of apoptotic tumor cells compared to control, which indicates that at the administered dose orlistat declined tumor load.

### 4: BMDM of orlistat-administered tumor-bearing mice display characteristic features of M_1_ subtype of Mϕ.

**Figure 6:**
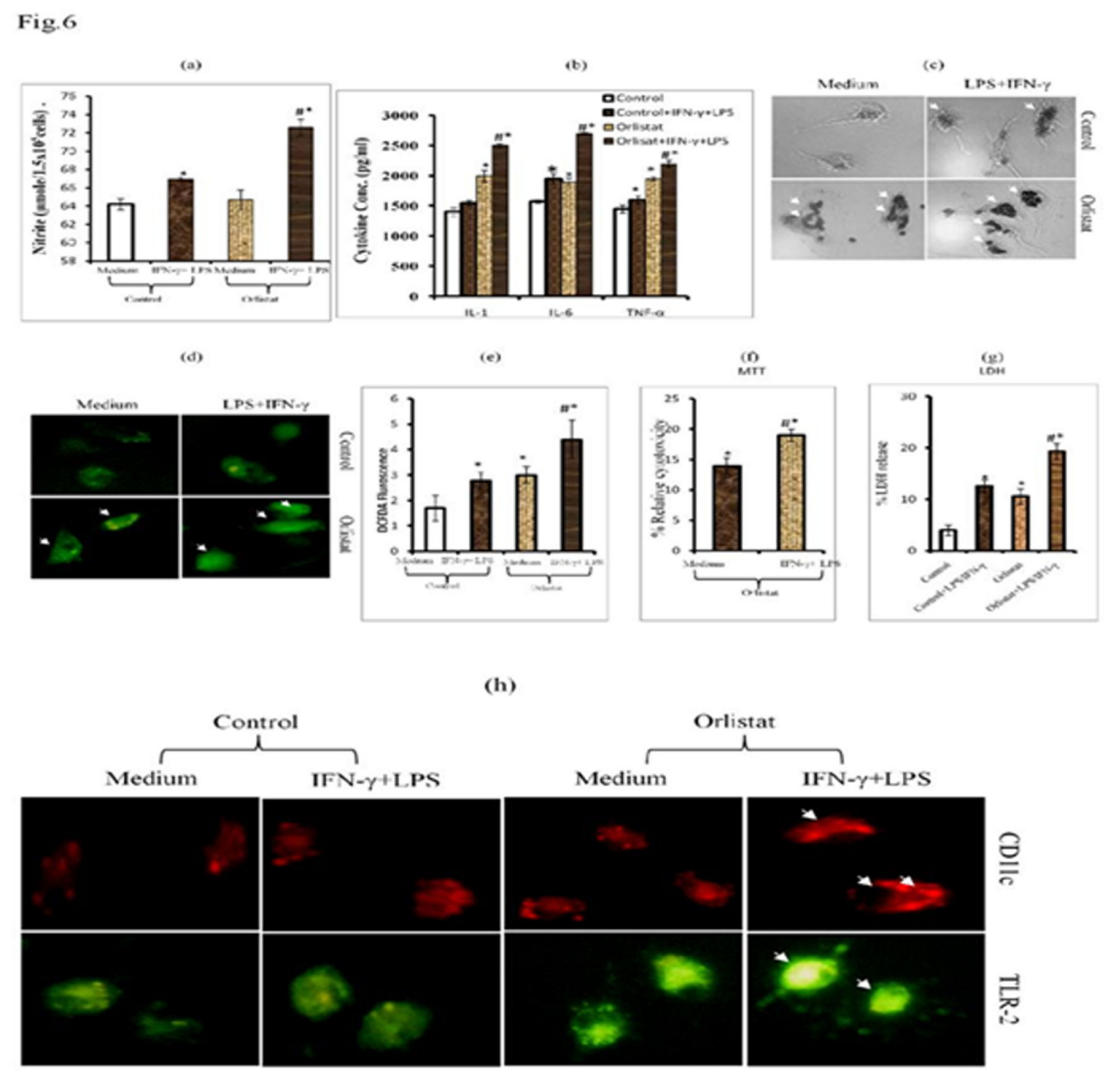
BMDM obtained from BMC of orlistat-administered groups display M1 Mϕ phenotype. BMDM differentiated from BMC of control or orlistat-administered tumor-bearing mice were incubated *in* *vitro* for 24h in medium alone or containing IFN- γ (100IU/ml) + LPS (10ng/ml) followed by estimation of NO (a), indicated cytokines by ELISA in culture supernatant (b), assay of ROS expression (d,e), phagocytosis (c), BMDM-mediated tumoricidal activity (f,g) and expression of cell surface functional markers: CD11c and TLR2 (h). Values shown in (a,b,e,f,g) mean ± SD of three independent experiments done in triplicate.*p<0.*05* *vs*. values of respective control. *#p*<*0.05 vs. values for orlistat and LPS + IFN-γ treated control groups. Arrows indicates increased phagocytosis (c), expression of ROS (d) and CD11c & TLR-2 (h) in BMDM of orlistat group treated with IFN-γ + LPS.

Considering the fact that BMDM differentiated from the BMC of orlistat-administered tumor-bearing hosts showed an augmented differentiation into macrophages, in the next part of the investigation we studied the responsiveness of these BMDM to activation signals of LPS and IFN-γ to determine their functional status and M_1_/M_2_ polarization. BMDM of control and orlistat-administered groups were incubated for 24 h in medium alone or containing LPS+IFN-γ followed by estimation of M_1_ macrophage markers: NO (Figure 6a), level of IL-1, IL-6 & TNF-α in the culture supernatant (Figure 6b), phagocytosis (Figure 6c), expression of ROS (Figure 6 d,e), tumor cytotoxicity (Figure 6f,g) and pattern of cell surface-associated functional proteins: CD11c & TLR-2 (Figure 6h). BMDM obtained from the BMC of orlistat-administered group showed a significantly increased production of NO along with IL-1, IL-6 and TNF-α in culture supernatent which was significantly up-regulated following *in vitro* treatment with IFN-γ + LPS. Similarly, the BMDM differentiated from the BMC of orlistat-administered group showed an augmented expression of ROS, phagocytosis and Mϕ-mediated tumoricidal activity, further enhanced upon treatment with IFN-γ + LPS. The expression of CD11c and TLR-2 was also found to be augmented in the BMDM of orlistat-administered group compared to control (Figure 6h).

### 5: Effect of orlistat on count of F4/80^+^ TAM

**Figure 7:**
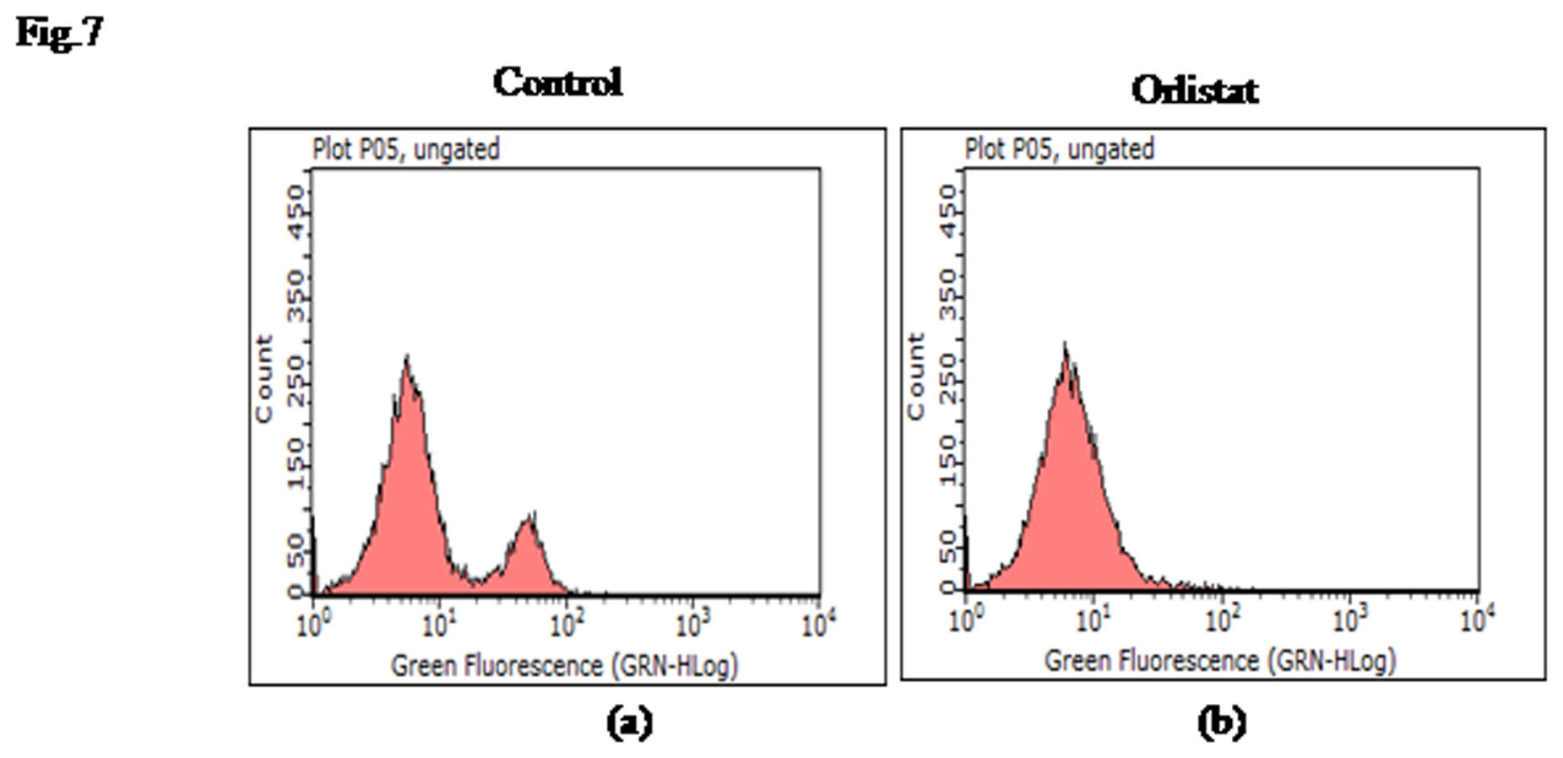
Enumeration of F4/80^+^ TAM following orlistat administration to tumor-bearing host. Peritoneal exudates cells obtained from control (a) and orlistat administered (b) tumor-bearing hosts were analysed for the number of TAM by flow cytometry using FITC conjugated antibody against macrophage marker F4/80. Data shown is from a representative experiment out of three independent experiments done in triplicate with similar results.

In view of the observations indicating that orlistat augmented macrophage differentiation, we also examined if orlistat administration modulated the number of F4/80^+^ TAM. The number of TAM declined in orlistat-administered group compared to untreated control (Figure 7 a,b).

## Discussion

In the present study the effect of orlistat administration on the process of myelopoesis in tumor-bearing hosts was examined. BMC harvested from orlistat-administered tumor-bearing hosts showed an enhanced differentiation into Mϕ displaying M_1_ phenotype in response to M-CSF. Next, we attempted to examine the possible mechanisms underlying myelopoietic action of orlistat in tumor-bearing hosts. The likelihood that orlistat could directly modulate the proliferative ability of BMC was, however, ruled out as *in vitro* treatment of BMC with orlistat at a dose range which manifest killing of tumor cells *in vitro*, did not alter their survival (data not shown). Thus it is likely that the myelopoietic action of orlistat on BMC in a tumor-bearing host of indirect nature. This proposition is further strengthened by previous reports which demonstrate that orlistat acts locally [13,21,46] and its systemic diffusion to distant anatomical locations, like bone marrow, is not well reported. Nevertheless, there is no report showing any harmful effect of orlistat on bone marrow cells in tumor bearing host. It is further reported that the action of orlistat varies depending on the fatty acid requirements of individual cell type [10–14,16,17,19–22]. Moreover, it is suggested that consequences of FASN inhibition may differ according to the stage of Mϕ differentiation [7]. 

The observed augmentary effect of orlistat on BMC survival and myelopoietic differentiation might also be dependent on tumor growth retardation. Indeed, in the present study we demonstrated that orlistat administration to tumor-bearing mice resulted in an augmented population of apoptotic tumor cells, indicating a declined tumor load. This is further corroborated in our another report demonstrating that administration of orlistat to DL-bearing hosts retarded tumor progression [47]. We and others have previously reported that orlistat induces tumor cell death [19–22,47]. Indeed, some previous reports from our and other laboratories have also indicated that reduction of tumor load *per se* is associated with an improved myelopoiesis [25–27,48–50]. As tumor growth and its inhibition are also associated with alteration in cytokine repertoire [20,25,27,47–49,51–53], the role of cytokine-dependent modulation of BMC survival and differentiation is not ruled out. This notion is supported by two lines of evidences: (1) our previous finding has shown that exposure of tumor cells to orlistat triggers a decline in production of IL-4 and IL-10 [20,47]. Indeed both of these cytokines have been reported to suppress haematopoiesis and Mϕ differentiation [54–62]: (2) Analyses of the serum cytokines pattern in orlistat-administered tumor-bearing hosts, as observed in this study, also showed a decline in the level of TGF-β and IL-10 along with elevation of IFN-γ. IFN-γ has been reported to potentiate myelopoiesis through multiple mechanisms [59–62]. Nevertheless, TGF-β has been reported to suppress myelopoiesis in tumor-bearing hosts [63–65]. Further, the findings of the present study also indicate that the BMC harvested from orlistat-administered hosts showed an inhibition in the induction of apoptosis along with modulation in the expression of cell survival regulatory. Moreover, we also observed an up-regulation in the expression M-CSF and M-CSFR, which have been reported to usher lineage specific Mϕ differentiation [54–58,61]. The expression of M-CSF and M-CSFR is in turn also regulated by cytokines [54–56,59–62]. This could be correlated to the findings of this study showing modulated cytokine level in serum of orlistat-administered tumor-bearing mice. Moreover, IFN-γ has been reported to modulate the expression of M-CSF and its receptor [40,42,44,60]. These cytokines are also implicated in modulating the expression of apoptosis regulatory Bcl2, Bax and Caspase-3 proteins [20,27,37,56,59–69]. On the basis of these correlations, it is suggested that the improved survival of BMC in tumor-bearing hosts, following administration of orlistat, could be attributed to prolonged BMC survival, enabling an adequate expression of receptors for cognate and non-cognate interactions leading to an augmented myelopoesis and differentiation of Mϕ. This notion is supported by the results showing that orlistat administration resulted in a rise in the population of macrophage precursors, indicating augmented macrophage differentiation.

**Figure 8:**
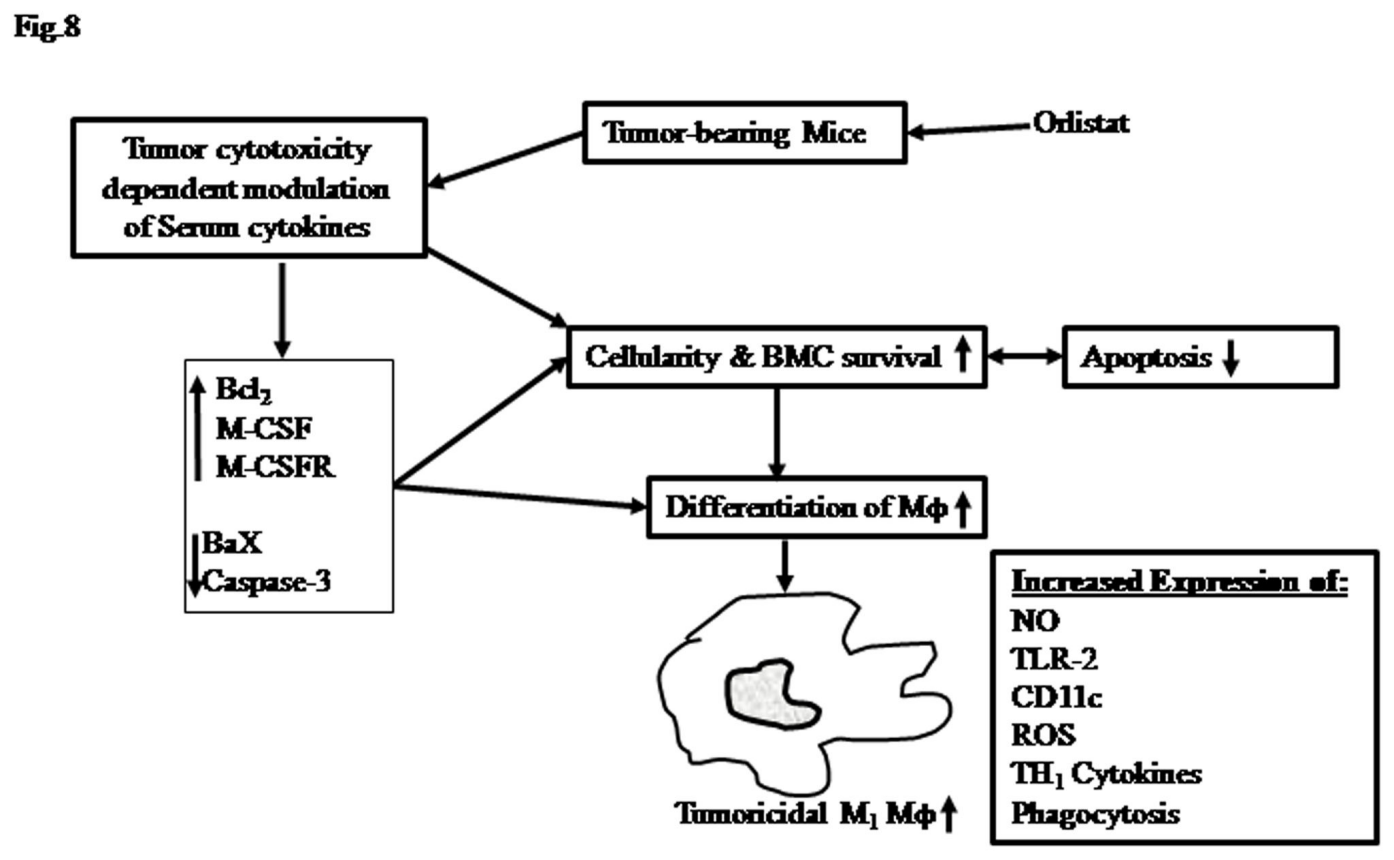
Summary of the suggested mechanisms underlying myelopoietic action of orlistat. Myelopoietic action of orlistat in tumor bearing hosts leads to augmented differentiation of Mϕ with M_1_ phenotype. Modulated expression of cytokines, cell survival and differentiation regulatory molecules play a central role.

We also observed that BMDM differentiated from the BMC of orlistat-administered tumor-bearing mice showed augmented phagocytosis, tumoricidal activity, expression of cell surface receptors like CD11c, TLR-2 and production of ROS, NO, IL-1 and TNF-α. These features indicate that these BMDM displayed a typical M_1_ Mϕ phenotype which is required for antitumor activity. Although orlistat is an inhibitor of FASN which is reported to be required by Mϕ for phagocytic ability and other activation associated functions [4–9], orlistat-administration to tumor-bearing mice did not inhibit the expression of FASN in BMC or BMDM (data not shown). The reason for this could be that orlistat poorly diffuses through systematic route to distant tissues [10,14,21,46]. These observations further reinforce the conclusion that the action of orlistat in augmenting the cell survival and differentiation of BMC in tumor-bearing hosts is of indirect nature. Interestingly, we observed that despite an augmentation in the differentiation of BMC precursors in macrophage lineage in orlistat-administered tumor-bearing hosts, the number of TAM showed a decline. This could possibly be due to the inhibitory action of orlistat on differentiation of TAM following their direct exposure to its therapeutic dose in the tumor microenvironment. Indeed, macrophages have been reported to be dependent on the activity of FASN for various membrane associated functions [7,8]. Moreover, other report also indicates the inhibitory action of FASN inhibitors on macrophage phenotype and their differentiation from monocytes [7]. However, the inhibitory action of orlistat on TAM in tumor microenvironment does not subdue its therapeutic value, as orlistat administration potentiated the differentiation of macrophages, which may help in augmenting antitumor immune responses, unlike other anticancer drugs which manifest immunosuppresion [1,70]. Summary of the proposed mechanism(s) underlying the myelopoietic action of orlistat in a tumor-bearing host is presented in Figure 8, indicating the role of modulated cytokine repertoire and expression of cell survival and differentiation regulatory molecules.

Taken together these findings indicate the worthiness of orlistat in a tumor-bearing host to inhibit tumor growth while also sustaining myelopoietic differentiation of tumoricidal macrophages. Conventional chemotherapeutics regimens on the other hand in general inhibit myelopoiesis. Thus the observations of the present study will be of immense help in further research on optimizing the use of orlistat for such dual benefits in tumor-bearing hosts. 
