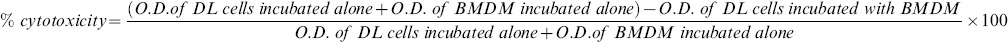# Correction: Myelopoietic Efficacy of Orlistat in Murine Hosts Bearing T Cell Lymphoma: Implication in Macrophage Differentiation and Activation

**DOI:** 10.1371/journal.pone.0093312

**Published:** 2014-03-24

**Authors:** The PLOS ONE Staff

Figure 1, Figure 6, and Equation 1 have been corrected for better readability.

**Figure 1:**
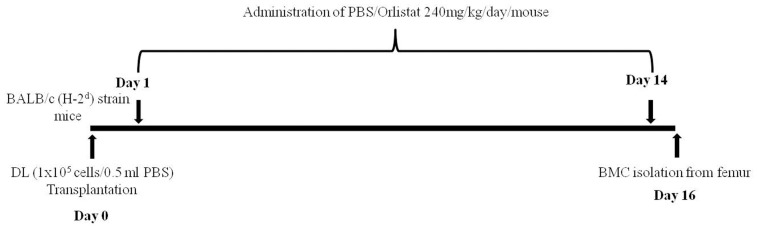
Protocol for administering orlistat to tumor-bearing mice. Mice were transplanted DL cells (1x105cells/0.5 ml PBS) on day 0 following administration of Vehicle alone (control) or containing orlistat 240mg/kg body weight/day up to day 14 post tumor transplantation. On day 16 BMC were harvested from femurs.

**Figure 6:**
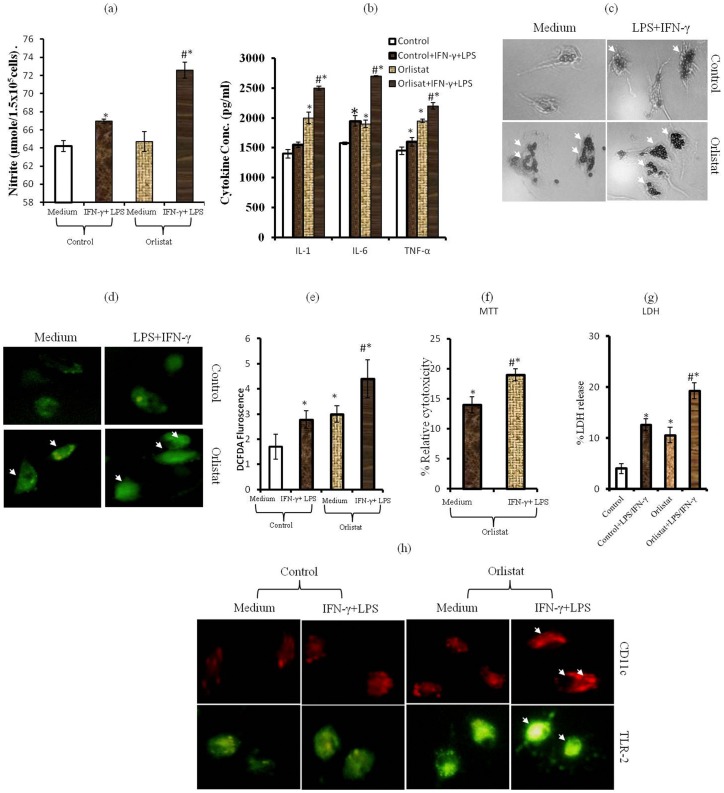
BMDM obtained from BMC of orlistat-administered groups display M1 Mφ phenotype. BMDM differentiated from BMC of control or orlistat-administered tumor-bearing mice were incubated *in* *vitro* for 24h in medium alone or containing IFN- γ (100IU/ml) + LPS (10ng/ml) followed by estimation of NO (a), indicated cytokines by ELISA in culture supernatant (b), assay of ROS expression (d,e), phagocytosis (c), BMDM-mediated tumoricidal activity (f,g) and expression of cell surface functional markers: CD11c and TLR2 (h). Values shown in (a,b,e,f,g) mean ± SD of three independent experiments done in triplicate.*p<0.*05* *vs*. values of respective control. *#p*<*0.05 vs. values for orlistat and LPS + IFN-γ treated control groups. Arrows indicates increased phagocytosis (c), expression of ROS (d) and CD11c & TLR-2 (h) in BMDM of orlistat group treated with IFN-γ + LPS.

Figure 1 is incorrect. The authors have provided a corrected version here.

Figure 6 is incorrect. The authors have provided a corrected version here.

Equation 1 is incorrect. The authors have provided a corrected version here.